# Extracellular Vesicles From *Sporothrix brasiliensis* Are an Important Virulence Factor That Induce an Increase in Fungal Burden in Experimental Sporotrichosis

**DOI:** 10.3389/fmicb.2018.02286

**Published:** 2018-10-02

**Authors:** Marcelo Augusto Kazuo Ikeda, José Roberto Fogaça de Almeida, Grasielle Pereira Jannuzzi, André Cronemberger-Andrade, Ana Cláudia Trocoli Torrecilhas, Nilmar Silvio Moretti, Julia Pinheiro Chagas da Cunha, Sandro Rogério de Almeida, Karen Spadari Ferreira

**Affiliations:** ^1^Department of Pharmaceutical Sciences, Institute of Environmental, Chemical and Pharmaceutical Sciences, Federal University of São Paulo, Diadema, Brazil; ^2^Department of Clinical Chemistry, Faculty of Pharmaceutical Sciences, University of São Paulo, São Paulo, Brazil; ^3^Department of Microbiology, Immunology and Parasitology, Federal University of São Paulo, São Paulo, Brazil; ^4^Special Laboratory of Cell Cycle, Center of Toxins, Immune Response and Cell Signaling, Butantan Institute, São Paulo, Brazil

**Keywords:** extracellular vesicles, *Sporothrix brasiliensis*, dendritic cells, virulence factor, sporotrichosis

## Abstract

Sporotrichosis is a mycosis that affects the skin, lymphatic system and other organs in humans and animals. The disease has a worldwide distribution, with endemic areas in Brazil, and is caused by a complex of species, including *Sporothrix brasiliensis*. Some fungi release extracellular vesicles (EVs) that can interact with the host cell and modulate the host immune response. The aim of this study was to analyze the participation of *S. brasiliensis* EVs in the modulation of dendritic cells (DCs) and in the control of infection *in vivo*. Our results showed that *in vitro*, the EVs isolated from *S. brasiliensis* induced an increase in the phagocytic index and fungal burden in DCs. In addition, we observed a significant increase in IL-12p40 and TNF-α cytokine production. Then, the EVs were inoculated into BALB/c mice before subcutaneous infection with yeast, and the lesion was analyzed after 21, 35, and 42 days. An increase in fungal burden and lesion diameter were observed after 21 days in mice inoculated with a high concentration of EVs. However, after 35 days, we observed a regression of the lesion, which persisted until 42 days after infection. Interestingly, we observed an increase in fungal burden in these mice. In addition, we observed the presence of immunogenic components and proteins that could be related with virulence in EVs. These results suggest that EVs can play an important role in virulence and modulation of the host immune system during experimental *S. brasiliensis* infection.

## Introduction

Fungal infections affect billions of people worldwide being a global concern for public health organizations (Gow and Netea, 2016). Sporothricosis, an infection caused by fungi species from *Sporothrix* genus, is a neglected disease present in several countries, with areas of high incidence in Brazil, China, and Africa (de Beer et al., 2016). In Brazil, *Sporothrix brasiliensis* is the main etiological agent of the human and animal sporothricosis (Silva et al., 2012; Dib et al., 2017) with over than 4,700 domestic felines and 4,000 humans cases reported in the last years (Barros et al., 2011; Silva et al., 2012; Pereira et al., 2014). Fungi infect skin and lymphatic system and may spread to other organs especially in an immunocompromised host (Moreira et al., 2015). *S. brasiliensis* has a high virulence profile in comparison with other similar species, which could be related to their secretion of specific virulence factors (Almeida-Paes et al., 2015; Della Terra et al., 2017).

Extracellular vesicles (EVs), including exosomes, microvesicles, and apoptotic bodies, are lipid bilayer structures released by cells in a diversity of organisms, which carry different components that might impact several biological processes (Yáñez-Mó et al., 2015). In fungi, EVs were first described in *Cryptococcus neoformans* (Rodrigues et al., 2007, 2008), and since then, were identified in several other pathogenic species, such as, *Histoplasma capsulatum* (Albuquerque et al., 2008), *Paracoccidioides brasiliensis* (Vallejo et al., 2011), *Sporothrix schenckii* (Albuquerque et al., 2008), *Candida albicans* (Vargas et al., 2015), *Malassezia sympodialis* (Rayner et al., 2017) and *Alternaria infectoria* (Silva et al., 2014). In addition, fungal EVs are associated with the transport of molecules across the cell wall along with the delivery of RNA, lipids, polysaccharides, proteins and immunoreactive components (Joffe et al., 2016; Rizzo et al., 2017). These EVs components can interact with host immune cells, contributing to drug resistance, and faciliting cell invasion and pathogenesis (Oliveira et al., 2010; Brown et al., 2015; da Silva et al., 2016). Moreover, during the infection process, the pathogen can release EVs that can circulate in body fluids and interact with different host cells, as shown in *C. neoformans*, where EVs were able to cross the blood-brain barrier, enhancing fungi pathogenesis in the brain (Huang et al., 2012).

Dendritic cells (DCs), specialized antigen-presenting cells, are an important innate component that recognizes pathogen patterns and can activate an adaptive response, inducing an immunity against the fungi (Verdan et al., 2012). EVs of pathogens can interact with DCs in different ways. EVs secreted by *Salmonella typhimurium* (Alaniz et al., 2007) were able to stimulate DCs, leading to the production of innate proinflammatory cytokines and stimulate DCs maturation. On the other hand, *Leishmania donovani* EVs inhibited production of cytokines and differentiation of Th1 cells by DCs (Silverman et al., 2010). In fungi, EVs from *C. albicans* were able to stimulate production of cytokines IL-12, IL-10, TGF-β and TNF-α by DCs, affecting host immune response against candidiasis (Vargas et al., 2015). Fungal EVs may represent an alternative strategy for treatment, once the role of their cargo are fully understood (Joffe et al., 2016).

Therefore, here, the aim of the present study was to analyze part of the composition of EVs from *S. brasiliensis* yeast cells, and their potential in modulating DCs and promoting protection against experimental sporotrichosis.

## Materials and Methods

### Fungal Strain and Culture Conditions

In all of the experiments, yeasts from *S. brasiliensis* strain 5110 (ATCC^®^ MYA-4823^TM^), provided by Professor Leila Maria Bezerra-Lopes that were originally isolated from cutaneous feline sporotrichosis, were grown on brain heart infusion (BHI) agar (Acumedia^®^, Neogen^®^) slants at 37°C for 7 days. For proteomic analysis, yeasts from *S. schenckii* strain M-64 (ATCC^®^ MYA-4822^TM^) grown under the same conditions as described above were used for comparison.

### Animals

Male BALB/c from 8 to 12 weeks old were obtained from the Center for Experimental Models Development for Medicine and Biology of the Federal University of São Paulo and housed in cages with *ad libitum* access to sterile food and filtered water in a temperature-controlled room at 23–25°C. The animal protocols and procedures used in this work were approved by the Ethics Committee and Animal Use (CEUA) from the Federal University of São Paulo^1^ (Protocol No. 3846300316).

### Preparation and Isolation of EVs

Extracellular vesicles were isolated as described (Oliveira et al., 2010; Vallejo et al., 2011). Briefly, yeasts were cultivated in BHI broth (BD Bioscience©) at 37°C under shaking at 150 rpm for 6 days. A cell-free supernatant was obtained by centrifugation at 4,000 × *g* for 15 min at 4°C and centrifuged at 15,000 × *g* for 15 min at 4°C to remove the debris. The supernatant was ultracentrifuged at 100,000 × *g* at 4°C for 1 h to sediment the EVs. The pellet was resuspended in sterile 1X PBS and ultracentrifuged again at 100,000 × *g* at 4°C for 1 h. The final pellet was resuspended in sterile 1X PBS and maintained at -80°C until use.

### Transmission Electron Microscopy (TEM)

Pellets containing yeast cells from culture for EV isolation were fixed with a solution containing 4% paraformaldehyde and 0.1% glutaraldehyde for 1 h at room temperature (RT). Fixed samples were washed and postfixed with osmium tetroxide, dehydrated in ethanol and embedded in spurr resin. Ultrafine sections were stained with uranyl acetate and lead citrate and examined by a transmission electronic microscope Tecnai^TM^ G2 F20 (FEI Company^TM^).

### Nanoparticle Tracking Analysis (NTA)

The size and concentration of the EVs were determined with the instrument NanoSight NS300 (Malvern Instruments Ltd.), a dark-field microscope equipped with a 405 nm laser and a sCMOS camera. Three 30-s videos were captured by a camera at level 13, with a viscosity adjusted for 0.9 cP and temperature at 25°C. Images were analyzed with NTA software version 3.1 (Malvern Instruments Ltd.).

### Sodium Dodecyl Sulfate-Polyacrylamide Gel Electrophoresis (SDS–PAGE) and Western Blotting Analysis

For SDS-PAGE gel electrophoresis and western blotting analysis, 50 μg of protein samples were loaded in 12% polyacrylamide gels. After electrophoresis, gels were silver-stained with silver nitrate (Silver Staining Kit - GE Healthcare©) or processed for western blotting. For comparison, an exoantigen from the yeast (de Almeida et al., 2014) was also analyzed. For western blotting analysis, proteins were transferred to nitrocellulose membranes (GE Healthcare©) using ECL Semi-Dry Blotter instrument (Amersham Biosciences^TM^). Membranes were blocked with 5% milk diluted in 1X PBS-Tween, for 2 h, washed with 1X PBS-Tween, and incubated overnight with total sera of uninfected mice, as negative control, or with sera of infected mice with *S. brasiliensis* in a ratio 1:200, or with 20 μg of monoclonal antibody P6E7, which recognizes gp70 (Nascimento et al., 2008). After incubation with primary antibodies, membranes were washed and incubated for 2 h with anti-mouse immunoglobulin linked to horseradish peroxidase (Bio-Rad^®^) in a ratio 1:5,000 for 2 h. Then, ECL substrate was added for 1 min and, in a dark room, membranes were incubated for 2 min with Amersham^TM^ Hyperfilm ECL for detection.

### Liquid Chromatograph-Coupled Tandem Mass Spectrometry (LC-MS/MS) Sample Preparation and Data Analysis

For LC-MS/MS analysis, 120 μg of protein samples were precipitated with 10 volumes of acetone and dissolved in 8 M of urea, followed by reduction and alkylation. Protein digestion was performed with Trypsin (Sigma-Aldrich^®^) in a ratio 1:20 of enzyme to substrate, at 37°C during 18 h and the reaction was stopped by addition of 5% of formic acid. Peptides were vacuum-dried and cleaned using Zip Tip micro-C18 (Millipore Merck©) before subsequent MS analysis. LC-MS/MS was performed as previously described (Leandro de Jesus et al., 2017). Briefly, peptides were resuspended in 0.1% formic acid and injected to a nano HPLC (NanoLC-1DPlus, Proxeon) coupled to an in-house made pre-column (5 cm × 10 μm) filled with a 10 μm C18 Jupiter^®^ resins (Phenomenex©). Peptides were fractionated on an in-house 10 cm × 75 μm reversed phase capillary emitter column filled with 5 μm C18 Aqua^®^ resins (Phenomenex©). The gradient of 2–35% of acetonitrile in 0.1% formic acid was performed by 60 min followed by a gradient of 35–95% for 8 min at a flow rate of 300 nl/min. The eluted peptides were directly analyzed in LTQ Orbitrap Velos (Thermo Scientific^®^). The source voltage and the capillary temperature were set at 1.9 kV and 200°C, respectively. The mass spectrometer was operated in a data-dependent acquisition mode (DDA) to automatically switch between one Orbitrap full-scan and 10 ion trap tandem mass spectra. The FT scans were acquired from m/z 200 to 2,000 with mass resolution of 30,000. The raw data were transformed to mgf files by using MS Converter software. Proteins were identified by searching against the Sporothrix database (downloaded at 2017 – 38,037 sequences) using MASCOT 2.4.1 version. Carbamidomethylation (C) was set as fixed modification while oxidation (M) and acetylation (N-terminal) as variable modifications; maximal missed cleavages of 2; MS1 tolerance of 10 ppm and MS2 of 0.8 Da. Protein lists were further analyzed using Gene Ontology pathway on Uniprot (access at December 2017)^2^.

### Isolation of DCs

Bone marrow-derived DCs were generated as previously described (Inaba et al., 1992). After mice euthanasia, femurs were isolated and flushed with Roswell Park Memorial Institute (RPMI) medium (J.T. Baker^®^). After isolation, bone marrow cells were cultivated in RPMI supplemented with 10% fetal calf serum (LCG Biotecnologia, Brazil) and EVs-depleted (ultracentrifuged at 100,000 *g* for 12 h), 10 μg/mL of gentamicin (Inlab, Brazil), and 50 ng/mL of recombinant mouse granulocyte macrophage colony-stimulating factor (GM-CSF; BD^®^) for 7 days at 37°C with 5% of CO_2_. On days 3 and 5, non-adherent cells were removed and fresh medium were added. On day 7, non-adherent cells were removed and analyzed in flow cytometer FACSCanto^TM^ (BD Bioscience©) with specific DCs cell surface markers, CD11c^+^ and MHC class II^+^.

### Trypan Exclusion Assay

Dendritic cells were cultivated in a 24-well cell culture plate at a concentration of 2 × 10^5^ cells/mL with EVs at different concentrations, EV1 (8 × 10^7^ EVs/well), EV2 (1.7 × 10^8^ EVs/well) and EV3 (3.5 × 10^8^ EVs/well), during 4 and 24 h at 37°C with 5% of CO_2_. Thereafter, the adherent cells were removed with a cell scraper and stained with 0.5% trypan blue stain (Invitrogen^TM^), followed by counting using an automated cell counter Countess^TM^ (Invitrogen^TM^), which measured trypan-negative, trypan-positive and total cells.

### Phagocytosis Assay and Analysis of Colony-Forming Units (CFU) From DCs

Dendritic cells at a concentration of 2 × 10^5^ cells/ml were cocultured with *S. brasiliensis* EVs at different concentrations, as described above, for 30 min in 24-well cell culture plates containing glass cover slips on their surface. After incubation time, cells were infected with yeasts at a ratio 1:1 for 4 h at 37°C with 5% of CO_2_. Next, cells were stained with a Wright stain (Laborclin, Brazil), and evaluated under a light microscope. The phagocytic index was calculated according to the following formula:

$$\mathrm{Phagocytic}\;\mathrm{index}=\left(\frac{\mathrm{Number}\;\mathrm{of}\;\mathrm{internalized}\;\mathrm{yeasts}}{\mathrm{Number}\;\mathrm{of}\;\mathrm{phagocytic}\;\mathrm{DCs}}\right)\times 100$$

For CFU analysis, DCs cultured with *S. brasiliensis* EVs under the same conditions as described above, were infected with yeasts for 24 h at 37°C with 5% of CO_2_. Next, the supernatant was removed, and the cells were lysed with sterile water. Lysates were diluted with PBS, plated on BHI agar plates and cultivated for 10 days at 37°C. Colonies from the plates were counted and calculated for average CFU per ml of cell lysate.

### *In vivo* Assays Infection

For analysis of the effect of *S. brasiliensis* EVs during *in vivo* infection, 3 groups of 15 animals received 2 doses of EVs at different concentrations, EV1 (8 × 10^7^ EVs/animal), EV2 (1.7 × 10^8^ EVs/animal) and EV3 (3.5 × 10^8^ EVs/animal) diluted in 100 μl of sterile 1X PBS via intramuscular injection at 7-day intervals. Positive and negative control groups were injected with PBS. Seven days after the second EV injection, the mice were infected subcutaneously with *S. brasiliensis*, as previously described (Castro et al., 2013) with slight modifications. For that, 1 × 10^7^ *S. brasiliensis* yeasts suspended in 100 μl of sterile 1X PBS were injected subcutaneously in the dorsal sacral region of animals in the EV and positive control groups. The negative group was only injected with PBS. Animals were monitored for 42 days to evaluate the progression of infection and for measurement of the average diameter of the skin lesion. Then, 21, 35, and 42 days postinfection, 5 animals from each group were euthanized, and organs were obtained for analysis.

### Analysis of Organ CFU and Histopathology

Skin lesions, liver, spleen, and lungs were weighed and macerated in sterile 1X PBS. Homogenized organs were diluted and plated in BHI agar plates for 10–15 days at 37°C. Colonies from the plates were counted and we calculated the average CFU per gram of organ. For histopathology, skin lesions were fixed in 10% buffered formalin for 48 h and then transferred into a solution of 70% ethanol. Fixed organs were processed and embedded in paraffin. Blocks were sectioned in 4-μm-thick sections, stained with Grocott methenamine silver (GMS) and analyzed under a light microscope. For GMS, a positive control slide was made to confirm the staining of the fungal structures.

### Cytokine Dosage

Supernatants from the DCs assays and organs macerated from the infected animals were centrifuged at 1,800 rpm for 10 min to remove the cell debris and analyzed for production of cytokines IL-1ß, IL-4, IL-6, IL-10, IL-12 p40, IL-12 p70, IL-17, TNF-α, and IFN-γ by Enzyme Immuno Linked Assay (ELISA) kits DuoSet^®^ (R&D System^®^) following the manufacturer’s instructions. Briefly, 96-well ELISA plates were coated with purified rat anti-mouse cytokine capture antibody overnight. Plates were washed with 1X PBS-Tween and blocked with PBS-1% BSA for 1 h. Samples or control cytokines (to develop a standard curve) were added overnight at room temperature. Plates were washed and incubated with biotinylated goat anti-mouse cytokine detection monoclonal antibody for 2 h. Then, streptavidin-horseradish peroxidase conjugate was added for 20 min, and development with substrate TMB was conducted for 20 min. The reaction was stopped with a solution of sulfuric acid 2 M, and absorbance was read in a microplate reader at 450 nm. The concentrations of the cytokines in the samples were calculated in comparison with the standard cytokine curve.

### Statistical Analysis

All statistics analysis were done by comparing data using One-way ANOVA analysis followed by Tukey test (Zar, 2010). All data are represented as the mean and standard deviation (SD).

## Results

### *Sporothrix brasiliensis* Secretes Extracellular Vesicles

**FIGURE 1:**
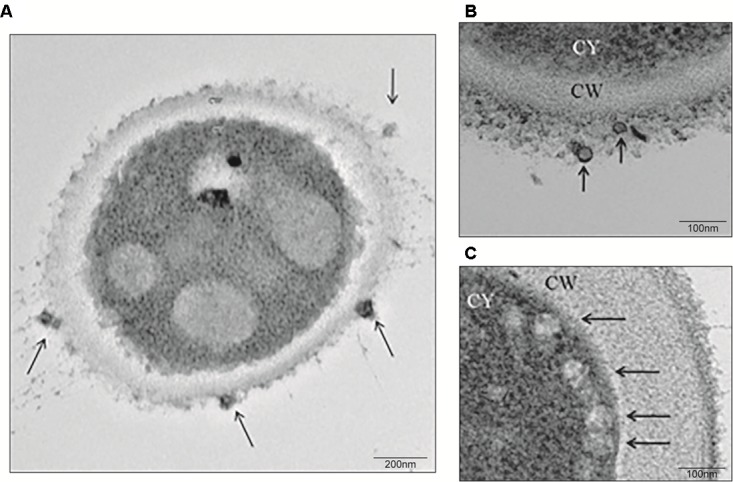
**Extracellular vesicle (EV)** release from *S. brasiliensis* yeasts. Yeasts were obtained from a culture of yeasts for 6 days in BHI broth and processed for TEM analysis. At a magnification of 29,000x **(A)** and 80,000x **(B,C)**, EVs (arrows) were observed at the surface of the cell wall (CW) **(A,B)** or secreted **(C)** from yeasts.

**FIGURE 2:**
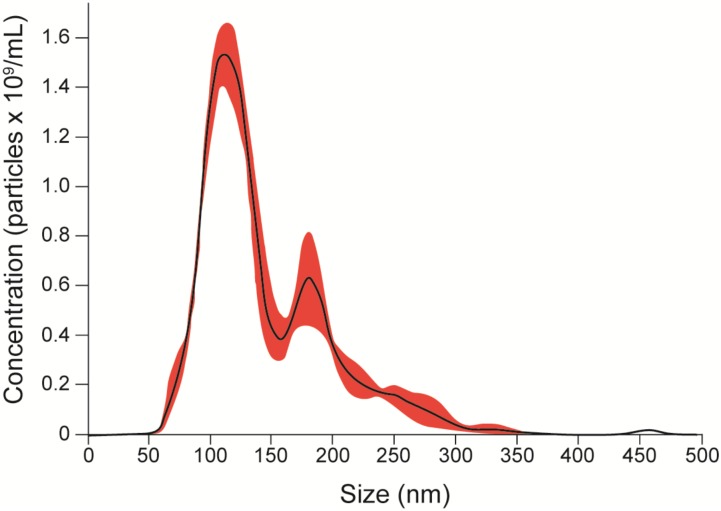
EV quantification. EVs were isolated by differential centrifugation of supernatant from a culture of yeasts for 6 days in BHI broth and analyzed by NTA. The graph shows the average concentration per size of EVs in samples (black), with a standard error of the mean (red). We obtained samples with a mean concentration of 1.19 × 10^11^ particles/mL with a mean size between 50 and 150 nm. Representatives from the average taken from the experiments are shown in three 30-s videos.

To evaluate the release of EVs, yeasts from *S. brasiliensis* were cultured in BHI broth for 6 days, and samples collected for TEM and NTA analysis. TEM analysis of fungi cells revealed EV-like structures in the cytoplasm, surface of the cell wall, and being secreted (**Figure 1**). NTA analysis of EV isolated from the supernatant of the culture by differential centrifugation, showed samples with EVs of a mean size ranging from 50 to 150 nm and with a mean concentration of 1.19 × 10^11^ particles/mL (**Figure 2**).

### Components of 70 and 100 kDa in EVs

**FIGURE 3:**
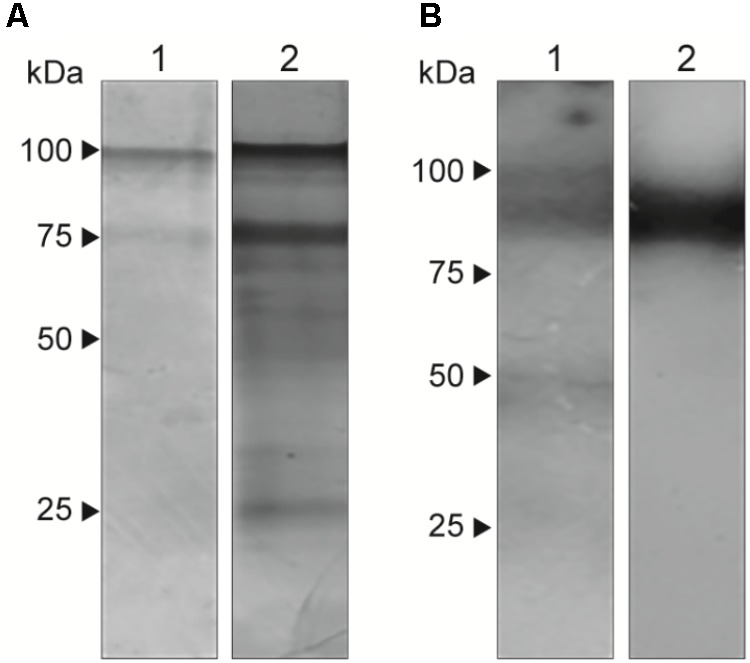
Presence of immunoreactive components in *S. brasiliensis* EVs. Analysis of EVs (1) in comparison with exoantigen (2) from yeasts. **(A)** SDS-PAGE gel stained with silver nitrate shows bands of 100 and 70 kDa. **(B)** Western blot with serum from infected mice with *S. brasiliensis* reveals a signal at approximately 100 kDa. The analysis was performed in three independent experiments.

Extracellular vesicles have different protein and components and are responsible for the transport of molecules to the external environment. Therefore, to analyze their composition, we performed SDS-PAGE (**Figure 3A**) to visualize 70-kDa and 100-kDa bands. In western blots (**Figure 3B**), EVs showed a different labeling pattern with total antibody from the sera of infected animals, with predominant reactivity in a double band at approximately 100 kDa. Additionally, we observed single bands at approximately 50 kDa and approximately 45 kDa. There was no reactivity with the monoclonal antibody P6E7 or with sera from uninfected animals (data not shown). Exoantigens from yeast were used to compare the composition of the EVs.

### EVs Increase the Phagocytic Index and Fungal Burden of DCs and Induce Cytokine Production

**FIGURE 4:**
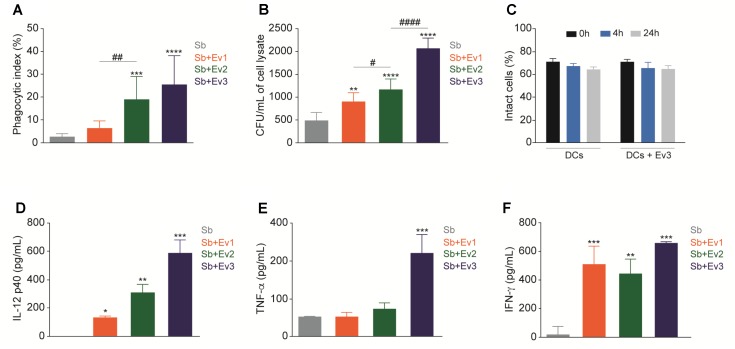
EVs increase phagocytosis and fungal burden from DCs. After stimulation with EVs for 30 min, DCs were cocultured with yeasts and presented an increase of phagocytic index after 4 h **(A)** and CFU after 24 h **(B)**. Intact cells were not altered when DCs were cultured only with EVs for 4 and 24 h **(C)**. After 24 h of coculture with yeasts, production of cytokines IL-12p40 **(D)**, TNF-α **(E)** and IFN-γ **(F)** increased in DCs stimulated with EVs. ^∗^*p* < 0.01, ^∗∗^*p* < 0.001, ^∗∗∗^*p* < 0.0001 compared with PBS. ^#^*p* < 0.01, ^##^*p* < 0.001, ^###^*p* < 0.0001 compared between different concentrations of EVs. The results are representatives from three independent experiments.

Dendritic cells are important in the recognition of pathogens and activation of the adaptive immune system. To verify the interaction of EVs in DCs, the cells were stimulated with EVs at different concentrations (EV1, EV2, and EV3) for 30 min, followed by coculture with *S. brasiliensis* yeast. After 4 h, a higher phagocytosis index was observed in the phagocytosis assay in DCs stimulated with EVs than in the unstimulated DCs (**Figure 4A**). However, after 24 h, upon CFU analysis, a higher CFU was obtained from cells stimulated with EVs than in those not stimulated (**Figure 4B**). The EVs did not alter the percentage of the intact cells when cultured with DCs for 4 or 24 h (**Figure 4C**). Cytokine dosages from all culture supernatants showed no production of IL-1β, IL-4, IL-6, IL-10, IL-12 p70, or IL-17 (data not shown). However, for IL-12p40 (**Figure 4D**), TNF-α (**Figure 4E**), and IFN-γ (**Figure 4F**), after 24 h, there was an increase in production in DCs stimulated with *S. brasiliensis* EVs that came into contact with *S. brasiliensis* yeast compared to that in non-stimulated DCs. There was no cytokine production associated with the interaction of DCs with *S. brasiliensis* EVs alone (data not shown).

### Skin Lesions and Fungal Load in a Murine Model Are Enhanced by EVs

**FIGURE 5:**
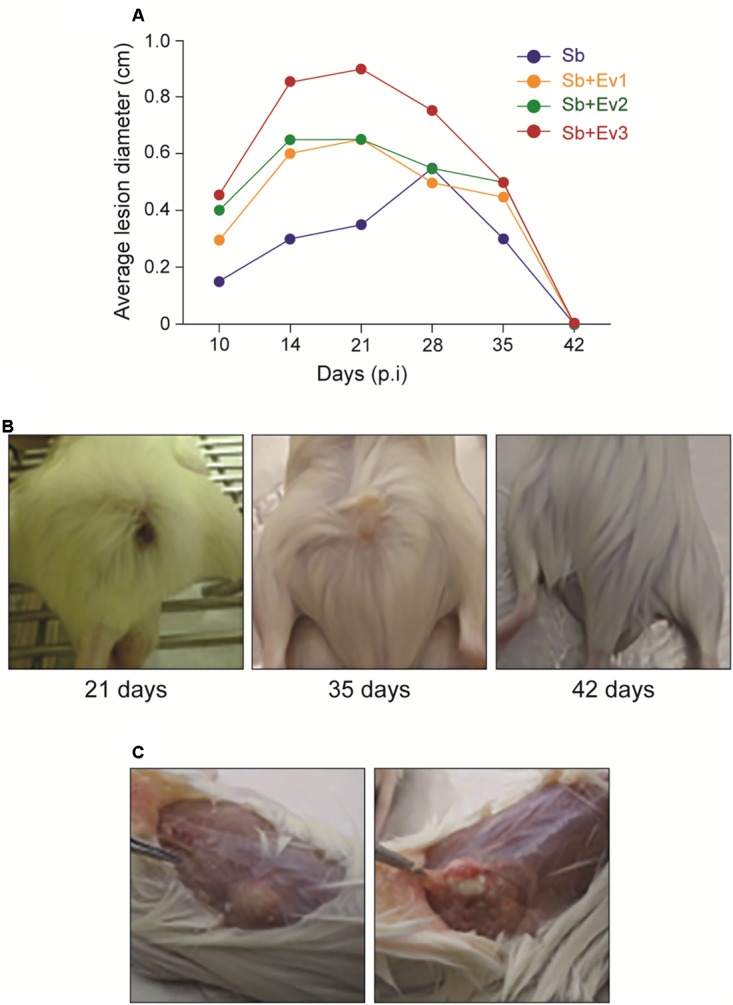
Progression of the subcutaneous infection in a murine model **(A)**. Graph represents the evolution of the average diameter of skin lesions for 42 days resulting from subcutaneous fungal infection. A representative of 3 independent experiments, containing 5 animals each group at each timepoint. **(B)** Figures represent the external appearance of skin lesions formed at the fungus inoculation site, with the presence of ulcerative nodular formation at 21 days, nodular healing at 35 days, and complete regression at 42 days. **(C)** Internal appearance of a skin lesion at 35 days. A subcutaneous (left) encapsulated formation is observed, which when cut contains a purulent exudate (right).

To verify the role of the *S. brasiliensis* EVs *in vivo*, a subcutaneous infection model with BALB/c mice was used. After fungi infection, a firm, non-adherent subcutaneous nodular lesion that ulcerated on the 10th day was observed in all animal groups. During the 21 days after infection, the average lesion diameter grew, and lesions from animals that received EVs prior to infection were larger than those among animals that did not receive EVs. However, around the 28th day, the lesions began to decrease in size, until they fully regressed by the 42nd day in all groups (**Figure 5**).

**FIGURE 6:**
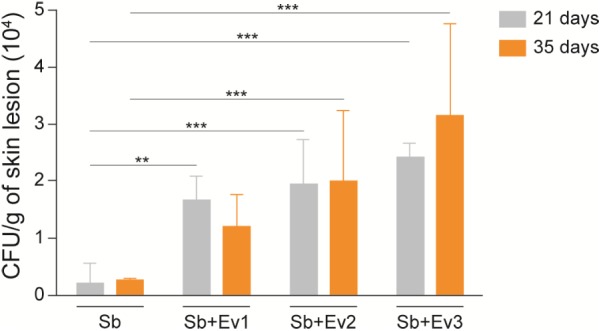
EVs increase the fungal load of a skin lesion in a murine model. From a skin lesion collected on the 21st and 35th day, CFU was analyzed. Animals that previously received EVs had a higher fungal burden than animals that did not receive EVs. A representative of 3 independent experiments, containing 5 animals per group, at each timepoint. One-way analysis ANOVA, with multiple comparisons by the Tukey test, was considered significant if *p* < 0.05. ^∗∗^*p* < 0.001, ^∗∗∗^*p* < 0.0001 compared with *S. brasiliensis* (positive control).

**FIGURE 7:**
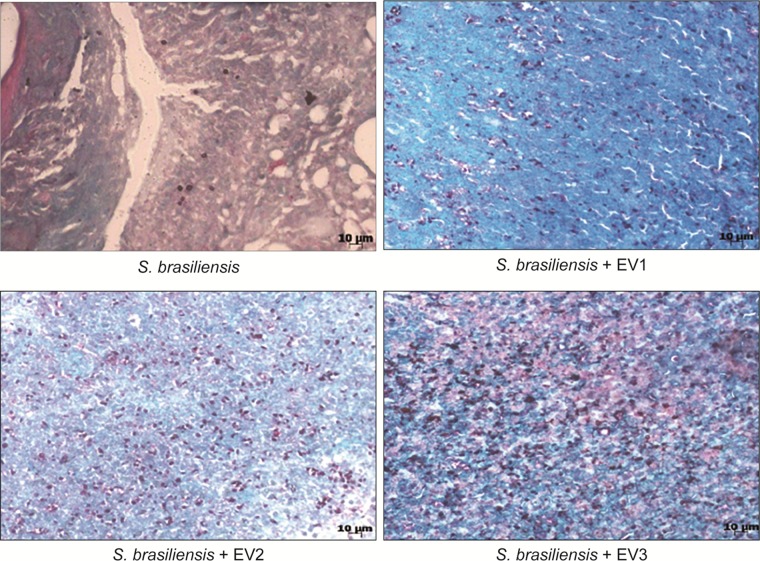
Histological analysis of a skin lesion after 21 days of infection. GMS stain, 40× magnification. We can observe the higher number of yeast structures stained in lesions of animals that received EVs before infection.

On the 21st, 35th, and 42nd day, five animals from each group were euthanized, and their organs were collected for analysis. At the CFU analysis, there was no fungal growth in the spleen, liver, or lungs at all time points. In the skin lesions on the 21st and 35th day (**Figure 6**), an increased fungal load was observed in animals that received EVs prior to infection in comparison to that in animals that had not received EVs. It was not possible to recover the fungus from the lesion sites on the 42nd day. Upon histological analysis by GMS staining, the same pattern was observed, with a higher fungal load in skin lesions of animals that received EVs before infection (**Figure 7**).

### Cytokine Production *in vivo*

**FIGURE 8:**
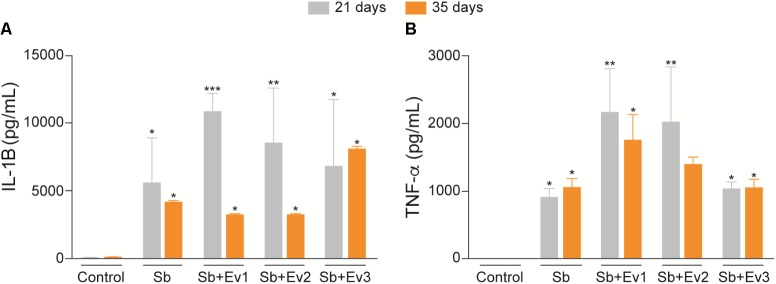
EVs induce production of cytokines in skin lesions of infected animals. In the presence of a fungus, there was an increase in IL-1ß **(A)** and TNF-α **(B)** levels in the skin lesion in comparison to the skin of uninfected animals. There was no difference in production of cytokines between infected groups stimulated with different concentrations of EVs. One-way analysis ANOVA, with multiple comparisons by the Tukey test, was considered significant if *p* < 0.05. ^∗^*p* < 0.01, ^∗∗^*p* < 0.001, ^∗∗∗^*p* < 0.0001 compared with negative control.

Cytokines are important in the immune response. Because of this, we studied the capacity of EVs in modulating the immunological response against *S. brasiliensis* infection. There was an increase in IL-1β and TNF-α production in the infected groups in relation to that in the uninfected group (**Figure 8**). However, administration of EVs did not alter IL-1β or TNF-α levels among the infected groups. There was no significant difference in IL-1β or TNF-α levels among the 21st, 35th, and 42nd day. In our results, no production of significant levels of cytokines were observed in the spleen, liver and lungs (data not shown). In the skin lesions, no production of IL-4, IL-6, IL-10, IL-12p40, IL-12p70 IL-17, and IFN-γ were observed (data not shown).

### Proteomics Analysis of EVs

**FIGURE 9:**
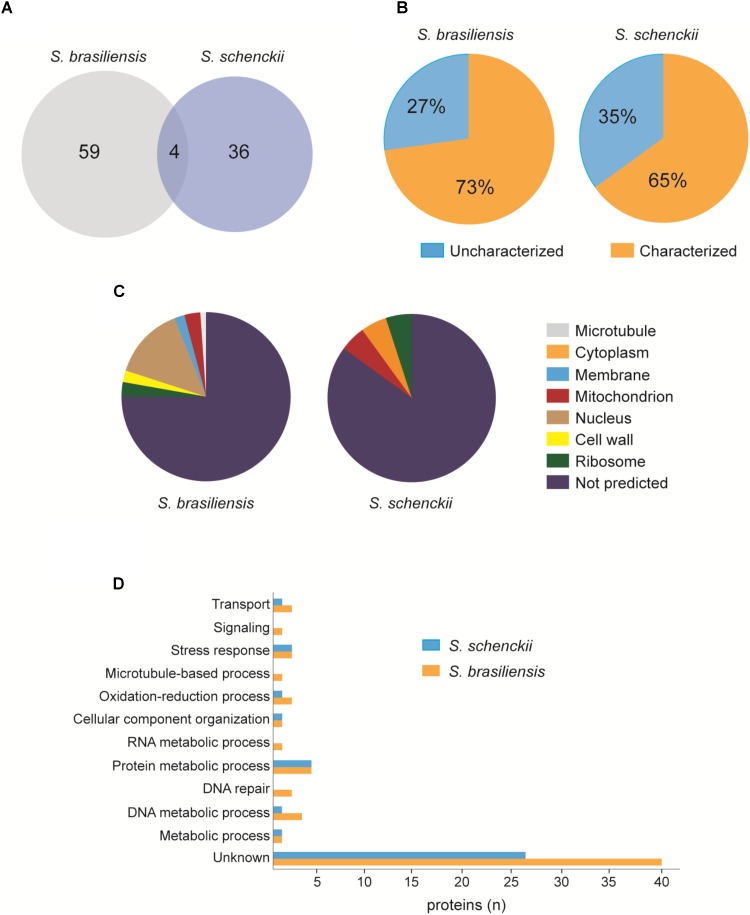
Proteomic analysis of EVs released from *S. brasiliensis* and *S. schenckii* **(A)**. Venn diagram representing the number of proteins found in both EVs. **(B)** Graphs representing the percentage of proteins characterized and not characterized in each EVs. **(C)** Representation of the predicted associated cellular component by GO analysis in each EVs. **(D)** Distribution of proteins according to the predicted biological process by GO analysis in each EVs.

To verify the protein composition of EVs, we performed LC-MS/MS. We found a total of 63 proteins in *S. brasiliensis* and 40 proteins in *S. schenckii* (**Supplementary Table 1**), where 4 proteins were common between the two species (**Figure 9A**). Analysis revealed that 27% of proteins in *S. brasiliensis* and 35% in *S. schenckii* were not characterized yet (**Figure 9B**). By gene ontology analysis, proteins were classified according to their predicted associated cellular component (**Figure 9C**) and biological process (**Figure 9D**).

## Discussion

Sporotrichosis is a mycosis that mainly affects the skin and lymphatic system and can spread to other organs. Disease severity depends on the virulence of the fungus and the immune response of the host. Fungi have been shown to be able to release EVs, which carry different types of molecules and can interact with host cells. The present work described the presence of EVs in the yeast phase of *S. brasiliensis*. After TEM and NTA analysis, *S. brasiliensis* was able to release EVs *in vitro*, as already described in *S. schenckii* (Albuquerque et al., 2008). In addition, we were able to analyze the composition of the EVs, finding 70-kDa and 100-kDa components with SDS-PAGE and on western blot, as already described in the analyses of exoantigens from *S. brasiliensis* (de Almeida et al., 2014; Della Terra et al., 2017). The 70-kDa protein is an important antigenic component, a virulence factor in sporotrichosis, described previously. Treatment with the monoclonal antibody (P6E7) made from this glycoprotein induces a reduction of fungal burden in the organs (de Almeida et al., 2014). However, the role of the 100-kDa component in EVs from *S. brasiliensis* is unclear. Our results suggest that EVs play a role in the transport of molecules produced by the fungus, some of which are recognized by the host’s immune system.

In *in vitro* assays, we observed an increase in phagocytosis by DCs stimulated with EVs, but viable yeast could be recovered from the cells. In addition, the DCs were not necrotic after their interaction with the EVs. These observations indicate that although cells engage in phagocytosis, they are not able to eliminate the fungus, unlike that observed in studies in murine macrophages with EVs from *C. neoformans* (Oliveira et al., 2010) and *P. brasiliensis* (da Silva et al., 2016), in which EVs from fungi increased the fungicidal capacity of the cells. Although the DCs stimulated with EVs from *S. brasiliensis* did not have a good fungicidal capacity, they could activate the immune system. In accordance with this, we observed an increase in IL-12p40 and TNF-α expression by DCs stimulated with different concentrations of EVs and after infection with *S. brasiliensis* yeast cells. These cytokines play very important immunoregulatory roles in host defense. Being pro and anti-inflammatory in nature, respectively, their functions are in opposition to one another. In experimental sporotrichosis, the role of the cytokines in the modulation of the disease is unclear.

After analyzing the potential of EVs to modulate DCs *in vitro*, we studied the capacity of the EVs to protect the mice against *S. brasiliensis*. BALB/c subcutaneous infection revealed that during the first 21 days and up to 35 days, the formation of the lesions was characteristic of sporotrichosis in all infected animals. However, in animals that previously received EVs, a greater mean diameter of lesions was observed. In addition, a higher fungal load was observed after CFU analysis and histological analysis of tissues from stimulated animals. Here, we observed that the greater the number of EVs injected into the mice, the greater the degree of fungal growth in the tissue. These results suggest that the administration of EVs favors the establishment of fungus in the skin. A similar result was observed in an *in vivo* infection by *C. neoformans*, where EVs favored the passage of fungus through the blood-brain barrier and increased its virulence in the central nervous system (Huang et al., 2012). After 35 days, skin lesions of animals from all groups began to decrease until total regression by 42 days. More studies are needed to understand the immune response in this period, but we know that, even after a clinical cure and lesion regression, cats need to continue their drug regimen to avoid relapse; therefore, the fungus may also remain in other tissues. In addition, when we looked at cytokine production, an increase in inflammatory cytokines IL-1ß and TNF-α production was also observed in the skin lesions of infected animals on days 21 and 35. A very intense inflammatory response may cause a change in the immune response, reducing fungus control and causing damage to adjacent tissue, which can facilitate the establishment of high fungal loads during these periods of infection (Morgado et al., 2011; Miranda et al., 2016).

By proteomic analysis, proteins related to several processes, such as metabolism and transport, were identified. These proteins can also act as virulence factors in infection, which could justify the intensification of the fungal load in our experiments. Heat shock proteins, described to be involved in the process of transition from mycelium to yeast in thermodimorphic fungi, such as *Histoplasma* sp. and *Paracoccidioides* sp., have great importance in adaptation to the host (Nimrichter et al., 2016; Cleare et al., 2017). Heat shock proteins have also been proven to be fundamental in the relationship between *C. neoformans* and cells in the immune system (Silveira et al., 2013). Major facilitator superfamily transporters are proteins responsible for the transport of molecules through the membrane. In *C. albicans*, deletion of genes related to this protein led to structural modifications that decreased its virulence (Shah et al., 2014). We have also found enzymes, such as serine/threonine protein kinases, that can alter proteins secreted by host cells, allowing the establishment of the pathogen (Canova and Molle, 2014). In addition, other enzymes such as cell wall glucanase (Nimrichter et al., 2016) can facilitate cell wall modifications that can enhance their immune system evasion capacity. It is worth to emphasize that we detected uncharacterized proteins, which can be related to virulence from fungi.

## Conclusion

Our results suggest that EVs from *S. brasiliensis* can carry important virulence factors and antigenic components that can have the capacity to interact with the immune response from the host and favor establishment by the fungus.

## Author Contributions

KF planned and wrote the study. MI did the development of the CFU, cytokine production, phagocyte assay, and histopathology. JA did the western blot. AC-A did the NTA. AT did the EVs analyzes. NM and JC performed the proteomic analysis. GJ, SA, AT, MI, and KF did the discussion. MI and KF discussed the results. All the authors reviewed the manuscript.

## Conflict of Interest Statement

The authors declare that the research was conducted in the absence of any commercial or financial relationships that could be construed as a potential conflict of interest.

## Footnotes

1 http://www.unifesp.br/reitoria/ceua/

2 http://www.uniprot.org/
